# The forbidden fruit – the thin line between belief, religion, and severe psychopathology: A case report

**DOI:** 10.1192/j.eurpsy.2021.1820

**Published:** 2021-08-13

**Authors:** F. Ferreira, I. Figueiredo, T. Ferreira, F. Viegas, N. Santos, C. Tomé, T. Maia

**Affiliations:** 1 Mental Health Department, Hospital Professor Doutor Fernando Fonseca, Lisboa (Amadora), Portugal; 2 Mental Health Department, Hospital Professor Doutor Fernando Fonseca, Lisboa (Amadora), Portugal; 3 Mental Health, Hospital Professor Doutor Fernando Fonseca, Lisboa (Amadora), Portugal

**Keywords:** Scrupulosity, Religion, obsessive–compulsive disorder, psychopathology

## Abstract

**Introduction:**

Religious obsessions constitute an interesting component of the phenomenology of obsessive-compulsive disorder(OCD). Scrupulosity can be phenomenologically similar to other OCD subtypes but the ultimate feared consequence is religious or moral in nature.

**Objectives:**

To develop a reflexion about religion, belief and its interaction with psychopathology, focusing on a clinical case.

**Methods:**

Review of the clinical case and literature.

**Results:**

37-year-old female patient with 4 prior psychiatric admissions. Stable until May 2020. After a brief online relationship patient develops subsequent guilt, anxiety and obsessive images with religious/sexual content. Abruptly, on the day of admission to the ER, the patient eats garlic in penitence and self-flagellate. At inpatient-unit she presented in mutism and total oral refusal, needing nasogastric tube for feeding and medication administration. She was medicated with diazepam and olanzapine, being added fluoxetine later on. In later interviews, a primordial idea based on the prevailing religious beliefs was found: “sex before marriage is a mortal sin”. This itself generated doubt “have I been forgiven” with compulsions of verification/purification (eg. repeated confession) and punishment, and this doubt almost reached a delirious character during the acute episode. Partial egodistonia, lived with suffering although with some continuity with her beliefs. At discharge patient showed insight for the unrealism of this dyad, though the primary idea remained immovable.

**Conclusions:**

Although the pharmacological approach managed to control the most disturbing symptoms presented by the patient, it’s worthwhile to review and to reflect on this report in a wider perspective, within in the light of the relevance to the clinical practice.

**Disclosure:**

No significant relationships.

